# Independent Supported Housing Versus Institutionalised Residential Rehabilitation for Individuals with Severe Mental Illness: A Survey of Attitudes and Working Conditions Among Mental Healthcare Professionals

**DOI:** 10.1007/s10597-022-01037-2

**Published:** 2022-10-13

**Authors:** Christine Adamus, Jovin Alpiger, Matthias Jäger, Dirk Richter, Sonja Mötteli

**Affiliations:** 1 Centre for Psychiatric Rehabilitation, Universitäre Psychiatrische Dienste Bern (UPD), Bern, Switzerland; 2 University Hospital of Psychiatry and Psychotherapy, University of Bern, Bern, Switzerland; 3 School of Applied Psychology, ZHAW Zurich University of Applied Sciences, Zurich, Switzerland; 4 Psychiatrie Baselland, Liestal, Switzerland; 5 Department of Psychiatry, Psychotherapy and Psychosomatics, Psychiatric Hospital of the University of Zurich, Zurich, Switzerland; 6 University of Zurich, Zurich, Switzerland; 7 Department of Health Professions, Bern University of Applied Sciences, Bern, Switzerland; 8 Centre for Psychiatric Rehabilitation, Universitäre Psychiatrische Dienste Bern (UPD), Sägestrasse 75, 3098 Köniz, Switzerland

**Keywords:** Independent Supported Housing, Housing rehabilitation, Residential care, Mental healthcare professionals, Attitudes, Severe mental illness

## Abstract

Despite widespread support for Independent Supported Housing (ISH) interventions, psychiatric housing rehabilitation still commonly takes place in residential care facilities (RCFs). This study compares preferences, attitudes and working conditions of mental healthcare professionals (MHCPs) in ISH and RCFs using an online survey. The survey included setting preferences, stress and strain at work, recovery attitudes, stigmatisation, and factors experienced as particularly important or obstructive in housing rehabilitation. Data were analysed using quantitative and qualitative approaches. Of the 112 participating MHCPs, 37% worked in ISH and 63% in RCFs. Professionals’ education, work-related demands and influence at work were higher in ISH, stigmatising attitudes were higher in RCFs. MHCPs in both settings endorsed ISH. The support process was seen as particularly important whereas stigmatisation, regulatory and political requirements were seen as obstructive for successful housing rehabilitation. Results indicate that social inclusion of individuals with severe mental illness is seldom feasible without professional support.

## Introduction

Adequate and stable housing conditions are a human right and a public health priority (World Health Organization WHO, 2021). Since deinstitutionalisation, housing rehabilitation has become an important component of psychosocial care for people with severe mental illness (SMI) and housing support needs (Farkas & Coe, 2019). In Western countries, various forms of housing rehabilitation have evolved. Traditionally, housing rehabilitation consists of a continuum of various institutionalised residential care facilities (RCFs), which are organised like a ‘stepladder’: individuals live in more or less intensively supported RCFs, and, as their level of functioning and housing skills improves, they move on to more independent living arrangements and eventually to their own apartments (Ridgway & Zipple, 1990). However, this linear continuum model does not work as intended, and a significant proportion of individuals remain in RCFs (Killaspy et al., 2019; Richter et al., 2016). In contrast, Independent Supported Housing (ISH) provides outreach housing rehabilitation based on the ‘Housing First’ approach and thus directly supports independent and autonomous living for people with SMI (Ridgway & Zipple, 1990). Under this approach, individuals live in their own apartments and are supported by a mobile team for an indefinite period of time.

ISH interventions clearly outperform institutionalised housing rehabilitation for homeless individuals in terms of their effectiveness, especially regarding increased housing stability and reduced hospitalisations (Tsai, 2020). However, with respect to non-homeless individuals, the current evidence does not reveal superiority of either housing rehabilitation setting (McPherson et al., 2018; Richter & Hoffmann, 2017a). Recent effectiveness studies suggest that ISH is similarly effective as or non-inferior to traditional RCFs (Dehn et al., 2022; Mötteli et al., 2022). Despite this, treatment guidelines recommend outreach support in an individual's own home as the first choice in housing rehabilitation, as it enables greater participation in the community and more self-determination for people with SMI than institutionalised RCFs (Gaebel et al., 2012; Gühne et al., 2019; World Health Organization WHO, 2021). The guidelines thus meet the requirements of the UN Convention on the Rights of Persons with Disabilities (UN CRPD) for social inclusion and the right to self-determination of people with SMI (United Nations, 2006). The lagging implementation of the UN CPRD (United Nations, 2022) is also reflected in the supply of housing rehabilitation services. Despite widespread support for ISH interventions, in many countries, the residential rehabilitation standard still consists of RCFs.

ISH is consistent with the preferences of individuals with SMI; most individuals strongly prefer independent living over residential institutions (Richter & Hoffmann, 2017b; Tanzman, 1993). In contrast to affected individuals, their family members as well as professionals tend to recommend assisted RCFs (Minsky et al., 1995; Piat et al., 2008). In a more recent study, caregivers' beliefs about the most appropriate living arrangements for their service users appeared to be so strong that they hindered the conduction of the study (Killaspy et al., 2019). Despite the clinical equivalence of institutionalised and outreach housing rehabilitation, many mental healthcare professionals (MHCPs) assumed different levels of support and supervision. However, neither these perceptions nor the degree of institutionalisation in the respective housing rehabilitation setting seem to reflect the severity of the service users’ illness and functional impairment (Valdes-Stauber & Kilian, 2015).

With the present study, we aimed to explore from a MHCPs’ perspective why the implementation of ISH interventions has been lagging despite the strong preferences among individuals with SMI and urgent recommendations from policies and treatment guidelines. For this purpose, we conducted a survey among MHCPs in outreach (ISH) and institutionalised (RCFs) housing rehabilitation settings and compared different work-related and attitudinal factors such as preferences for either housing rehabilitation approach (‘continuum’ vs. ‘Housing First’ approach), attitudes toward mental health and recovery, as well as working conditions or job demands between MHCPs working in ISH and RCFs. In addition, we aimed to examine obstacles MHCPs face and factors they perceive as important for successful housing rehabilitation of service users and to compare these between the two settings.

## Methods

An online survey was conducted among MHCPs in psychiatric housing rehabilitation settings in the three largest German-speaking cities in Switzerland: Bern, Basel and Zurich. All three cities offer ISH interventions and different RCFs, such as residential care homes, and assisted/supported group homes for people with SMI (see Table 1). Heads of facilities and services were targeted via email for participation. In October 2021, all MHCPs were asked to participate in a survey on challenges and attitudes concerning their work on housing rehabilitation for individuals with SMI. The survey was conducted in German and took 10–20 min to complete. Responses were collected anonymously using Unipark survey software. A total of 117 MHCPs responded to the survey, giving an estimated response rate of 33%. Of these, five persons were excluded from the analyses (four did not belong to a caregiving group, and one subject had many missing values, including the rehabilitation setting). The survey included questions concerning MHCPs’ preferences, attitudes, stress and strain at work, factors perceived as obstructive or important for successful housing rehabilitation and sociodemographic variables. Ethical approval was not required as confirmed by the Ethics Committee of the Canton Zurich, Switzerland (Req-2022-00714).

**Table 1:** Overview of participating housing rehabilitation services

Independent Supported Housing (ISH)	*N* (%)	Residential care facilities (RCFs)	*N* (%)
Basel	24 (35%)		44 (65%)
Verein für Sozialpsychiatrie Baselland		Verein für Sozialpsychiatrie Baselland	
Stiftung Rheinleben		Stiftung Rheinleben	
Inclusioplus		Inclusioplus	
Bern	13 (43%)		17 (57%)
WohnAutonom UPD		Wohnverbund UPD	
Zürich	5 (36%)		9 (64%)
Wohn-Coaching PUK		Zürcher Wohn- und Arbeitskoordination	
Total (*N* = 112)	42 (37.5%)		70 (62.5%)

*UPD* Universitäre Psychiatrische Dienste Bern*; PUK* Psychiatrische Universitätsklinik Zürich*; N* Sample size

### Variables and Instruments

All the MHCPs were asked for information on their gender, age, education (1: no completed schooling to 6: university or technical college), professional qualification, employment level, number of years working in psychiatric housing rehabilitation, rehabilitation setting (ISH or RCFs), and place of work (Basel, Bern, or Zurich).

Psychological stress and strain at work were measured using the Copenhagen Psychosocial Questionnaire (COPSOQ) (Kristensen et al., 2005; Nübling et al., 2006). In this study, three subscales were assessed using a 5-point scale: ‘emotional and quantitative demands’ (15 items, 1: very high demands to 5: very low demands; *m* = 3.08, *SD* = 0.68, Cronbach’s α = 0.81); ‘influence and development’ (12 items, 1: very high influence to 5: very low influence; *m* = 2.07, *SD* = 0.47, Cronbach’s α = 0.83); and ‘job strain’ (18 items, 1: no strain to 5 high strain; *m* = 2.22, *SD* = 0.45, Cronbach’s α = 0.84). Job demands and possibilities to take influence are assumed to be related with job strain, which is defined as the effects of job characteristics on satisfaction and health (Nübling et al., 2006).

Implicit theories of mental health were measured using an adapted scale from Schreiber et al. (2020). This scale uses a 7-point scale (1: strongly disagree to 7: strongly agree) to assess the extent to which MHCPs perceive mental health as malleable versus stable. Higher scores indicate higher agreement for mental health as a malleable trait (*m* = 5.53, *SD* = 0.95, Cronbach’s α = 0.84). An incremental theory of mental health is considered as essential when working with persons with SMI toward functional goals or recovery (Schreiber et al., 2020).

Attitudes toward recovery from psychiatric disorders were assessed using the Recovery Attitudes Questionnaire (RAQ-7) (Borkin et al., 2000; Jaeger et al., 2013; Rabenschlag et al., 2012). The questionnaire was validated for both patients/service users and health-care professionals. The 7-item scale assesses recovery attitudes based on a 5-point scale (1: strongly disagree to 5: strongly agree), with higher scores indicating a more positive attitude toward recovery (*m* = 4.21, *SD* = 0.45, Cronbach’s α = 0.68). The scale can be divided into the subscales of ‘recovery is possible’ and ‘recovery is difficult and differs among people’. A positive recovery orientation is associated with the assumption that meaningful goals can be achieved despite the presence of psychiatric symptoms (Anthony, 1993) and with more process- and person-oriented working toward rehabilitation goals (Rabenschlag et al., 2012).

To assess the extent of stigmatisation of MHCPs toward their service users, attitudes toward individuals with mental illness were assessed using the Opening Minds Stigma Scale for Health Care Providers (OMS-HC) (Modgill et al., 2014; Zuaboni et al., 2021). The OMS-HC includes 15 items to be answered on a 5-point scale (1: strongly disagree to 5: strongly agree) with higher scores indicating more negative attitudes toward persons with mental illness (*m* = 1.81, *SD* = 0.41, Cronbach’s α = 0.72). The scale includes the three subscales ‘attitudes’, ‘social distance’ and ‘disclosure’.

Preferences for a certain housing rehabilitation approach were surveyed using the following statements on a 10-point scale (1: strongly disagree to 10: strongly agree). The statements were developed based on expert opinions and were pretested and discussed with three MHCPs. Agreement with a statement is assumed from a value equal or greater of 6. The first statement refers to the general importance of housing rehabilitation in the treatment process, the second statement addresses the traditional continuum approach, and the third statement focuses the ‘Housing First’ rehabilitation approach (see Table 2):

**Table 2:** Working conditions and attitudes of MHCPs in outreach and institutionalised housing rehabilitation settings

	Independent Supported Housing (ISH; *N* = 42)		Residential care facilities (RCFs; *N* = 70)		
Variables	*m*	*SD*	*m*	*SD*	*p*
COPSOQ subscales					
Emotional and quantitative demands	2.84	0.72	3.22	0.62	.004
Influence and development	1.87	0.36	2.19	0.49	< .001
Job strain	2.16	0.38	2.26	0.48	.240
Implicit theory: malleable mental health	5.60	0.92	5.48	0.97	.512
Attitudes toward recovery (RAQ-7)	4.30	0.42	4.16	0.47	.097
Attitudes toward persons with SMI (OMS-HC)	1.68	0.36	1.89	0.41	.008
Preferences for a certain housing rehabilitation approach					
Importance of stable housing	5.69	2.60	5.36	2.36	.244
Continuum approach	3.26	2.37	5.51	2.64	< .001
‘Housing First’ approach	7.48	2.04	5.71	2.27	< .001

COPSOQ Copenhagen Psychosocial Questionnaire (Scale 1–5, 1 = higher demands, more influence, less strain); Implicit theories (Scale 1–7); RAQ-7 Recovery Attitudes Questionnaire (Scale 1–5); OMS-HC Opening Minds Stigma Scale for Health Care Providers (Scale 1–5); Preferences for a certain housing rehabilitation approach: Scales 1–10; for all scales except COPSOQ: higher values indicate stronger agreement. Differences between settings were tested using two-sample t-tests

General importance of stable housing:

1. “I believe that achieving stable housing should be prioritised over psychiatric/ psychological treatment for individuals with SMI.”
  Continuum approach:
2. “I believe that individuals with SMI need to first improve their living skills in a protective setting before they are able to return to independent living.”
  ‘Housing First’ approach:
3. “I believe that individuals with SMI best acquire housing skills while living independently with outreach support.”
  Factors perceived as important or as obstructive in terms of the participants’ successful housing rehabilitation were surveyed using the following two open questions:
4. “What aspects do you consider most important in enabling individuals with SMI to live equitably and stably?”
5. “What hinders you most in fulfilling your job tasks, for example supporting service users in improving their housing skills and finding a more independent housing form?”

### Data Analysis

Differences between the three locations regarding the rehabilitation setting and sociodemographic variables were tested using the Kruskal–Wallis and chi-square tests. Differences in MHCPs’ attitudes and stress and strain at work between the ISH and RCFs rehabilitation settings were tested using two-sample t-tests. To control for different sample characteristics in education level (see below), general linear models were calculated with dichotomised education levels (university degree versus others). The qualitative responses to the open questions were analysed using the thematic analysis approach by Braun and Clarke (2006). The first and last authors coded the responses independently. The codes were then compared and non-matching codes were revised. In a next step, the same authors independently summarised the codes into superordinate themes. These themes were then compared for agreement and revised after a consensus discussion. The frequencies of these themes between ISH and RCFs were tested using chi-square or Fisher exact tests. All the statistical analyses were performed using SPSS for Windows (version 28) with a 5% significance level.

## Results

### Sample Description

Among the 112 participating MHCPs, 62.5% (*n* = 70) were female, 36.6% were male (*n* = 41), and 0.9% (*n* = 1) selected the ‘diverse’ category. The mean age was 40.3 years (*SD* = 11.8), and 75.9% of the MHCPs had completed higher education (higher vocational school, university). Most were employed in social pedagogy (68.8%) or care (9.8%), 9.8% were working in management positions, and 11.6% indicated other designations, such as peer worker or assistant functions. The employment hours level ranged from 30 to 100%, with most (72.3%) working between 60 and 80%. The average length of employment in the current profession was *m* = 9.6 (*SD* = 8.6) years. At the time of the survey, 62.5% were working in RCFs and 37.5% in ISH. There were no significant differences in the sample characteristics between the Basel, Bern, and Zurich sites.

### Outreach Versus Institutionalised Housing Rehabilitation Settings

The participating MHCPs from outreach and institutionalised housing rehabilitation settings did not differ regarding their sociodemographic characteristics (gender, age, professional qualification, employment level, and work experience), except with regard to their education. In ISH, 90.5% of the MHCPs reported having completed higher education (59.5% with university degree), whereas this was only true for 67.1% of those working in RCFs (27.1% with university degree; *p* < 0.05).

### Working Conditions and Attitudes Toward Mental Health

The mean scores in terms of the assessed instruments are shown in Table 2 for both housing rehabilitation settings. Stress and strain at work (COPSOQ) were in the mid-range overall. Emotional and quantitative demands (subscale 1), as well as influence and possibilities for development at work (subscale 2), were significantly higher in ISH than in RCFs. Job strain, which included the effects on one’s health and satisfaction level (subscale 3), showed no significant differences between the settings. In line with the theory underlying the COPSOQ (Nübling et al., 2006), job strain was correlated with job demands (*r* = − 0.32, *p* < 0.001, *n* = 112; higher demands: more negative effects) and with the influence and development subscale (*r* = 0.56, *p* < 0.001, *n* = 112; higher influence: more positive effects). For all three subscales, the same results were also obtained after controlling for education.

The majority of the MHCPs in both settings accepted, to a similar extent, the implicit theory of mental health as a malleable trait, with no significant differences between the settings. MHCPs with higher scores on this scale also showed more positive attitudes toward recovery (*r* = 0.37, *p* < 0.001, *n* = 112). Recovery attitudes were similar in both settings with no significant differences. MHCPs from ISH had less stigmatising attitudes toward individuals with mental illness than participants from RCFs (lower scores on the OMS-MH; *p* < 0.01); this difference remained significant after controlling for education.

The items on preferences for a certain housing rehabilitation approach received medium agreement in both settings regarding the general importance of stable housing versus psychiatric treatment indicating that MHCPs perceive their actual work as important in the rehabilitation process of their service users. The traditional continuum model of housing rehabilitation obtained significantly more agreement in RCFs than in ISH; for the ‘Housing First’ approach, the opposite pattern was observed. In absolute numbers, the ‘Housing First’ approach received higher approval than the continuum approach in both settings (see Table 2). Totally, 63.3% of the MHCPs endorsed ISH interventions (scores ≥ 6; RCFs: 54.4%; ISH: 78.6%).

### Supportive and Obstructive Factors

Overall, the same factors were perceived as particularly important for successful housing rehabilitation under both settings (Table 3). Characteristics of the support process were stated most frequently. Low-threshold access to the required services and recovery-oriented MHCPs are considered especially important. Service users’ characteristics were seldom named as important factors for successful housing rehabilitation.

**Table 3:** Particularly important factors for successful housing rehabilitation of persons with SMI

	Independent Supported Housing (ISH; *N* = 41)		Residential care facilities (RCFs; *N* = 70)		
Categories from qualitative data analysis (open question format; multiple answers possible)	*n*	%	*n*	%	*p*
Characteristics of service users	3	7.3	10	14.3	0.366
Characteristics of the support process	35	85.4	55	78.6	0.378
Characteristics of the social environment	16	39.0	22	31.4	0.416
Contextual circumstances	13	31.7	22	31.4	0.976
Single codes					
Characteristics of service users					
Stable mental health condition	0	0.0	4	5.7	
Competencies and resources (incl. motivation)	2	4.9	6	8.6	
Compliance/adherence to therapeutical or medical interventions	1	2.4	3	4.3	
Characteristics of the support process					
Low-threshold support offers	17	41.5	28	40.0	
Transparent and shared goals	4	9.8	7	10.0	
MHCPs’ resources and time/content flexibility	4	9.8	8	11.4	
MHCPs orientation/focus on recovery, resources, and relationship	18	43.9	25	35.7	
Networking with authorities, departments, therapists, etc	6	14.6	5	7.1	
Characteristics of the social environment					
Support from social environment (private, neighbourhood, work & everyday life)	11	26.8	16	22.9	
Comprehension, acceptance, support and inclusion by society	7	17.1	7	10.0	
Contextual circumstances					
Affordable accommodations/suitable housing conditions (incl. RCF *n* = 1)	9	22.0	12	17.1	
Financial resources of service users	2	4.9	4	5.7	
Appropriate (supervised) jobs, services for daily activities	3	7.3	9	12.9	
Ensured funding of support services	1	2.4	1	1.4	

Tests were performed with chi-square or Fisher exact tests

*N* Sample size; *MHCPs* Mental healthcare professionals; *RCF* Residential care facility

Regarding the factors perceived as obstructive in successful housing rehabilitation, some differences between the settings appeared (Table 4). In ISH, contextual circumstances such as the lack of affordable housing, service users’ financial resources and regulatory and political requirements, were most frequently stated as factors that hinder rehabilitative work, followed by characteristics of the social environment and social stigmatisation in particular. In RCFs, contextual circumstances, characteristics of the social environment, and service users’ characteristics (poor health, limited resources and motivation, unrealistic self-perception) were mentioned with similar levels of frequency. Two answers regarding suitable housing conditions referred to the working context in an institution, such as the structural properties of the residential facility.

**Table 4:** Obstructive factors in housing rehabilitation of persons with SMI

	Independent Supported Housing (ISH; *N* = 40)		Residential care facilities (RCFs; *N* = 69)		
Categories from qualitative data analysis (open question format; multiple answers possible)	*n*	%	*n*	%	*p*
Characteristics of service users	8	20.0	31	44.9	0.009
Characteristics of the support process	14	35.0	30	43.5	0.385
Characteristics of the social environment	18	45.0	17	24.6	0.028
Contextual circumstances	30	75.0	32	46.4	0.004
Single codes					
Characteristics of service users					
Inability to live independently	1	2.5	5	7.2	
Severe impairment, high support needs	2	5.0	4	5.8	
Poor mental health	2	5.0	7	10.1	
Limited personal resources and skills, lack in motivation, resignation, self-stigmatisation	6	15.0	14	20.3	
Unrealistic self-assessment	0	0.0	9	13.0	
Characteristics of the support process					
Problems in setting (common) goals or clarifying tasks/lack in compliance	5	12.5	11	15.9	
Lack in MHCPs’ resources and time/content flexibility	8	20.0	16	23.2	
Discrepancies in the helper system	1	2.5	4	5.8	
Insufficient cooperation with external specialists	1	2.5	1	1.4	
Characteristics of the social environment					
Lack in social support (e.g. absent or negative social environment)	4	10.0	4	5.8	
Social stigmatisation	14	35.0	14	20.3	
Contextual circumstances					
Lack in affordable accommodations/suitable housing (incl. RCF: *n* = 2)	16	40.0	14	20.3	
Limited financial resources of service users	14	35.0	8	11.6	
Requirements and general conditions of authorities an politics	15	37.5	8	11.6	
Administrative workload	3	7.5	4	5.8	
Internal problems at the workplace	1	2.5	4	5.8	

Tests were performed with chi-square or Fisher exact tests

*N* Sample size; *MHCPs* Mental healthcare professionals; *RCF* Residential care facility

## Discussion

Most of the 112 participating housing rehabilitation MHCPs from ISH and RCFs for individuals with SMI favour the ‘Housing First’ approach over the conventional continuum of different RCFs. The so far tentatively implemented ISH interventions therefore not only correspond to the preferences of individuals with SMI (Richter & Hoffmann, 2017b) and to current policies (United Nations, 2006) and guidelines (Falkai, 2012; Gaebel et al., 2012; World Health Organization WHO, 2021), but also receive endorsement from MHCPs in both settings. The MHCPs’ preferences for a certain housing rehabilitation approach in general does not seem to contribute to the lagging implementation of ISH interventions. Also, MHCPs’ attitudes toward recovery did not show relevant differences between the settings. However, MHCPs’ attitudes in RCFs showed higher levels of stigmatisation toward persons with SMI. In addition, RCF workers mentioned service users’ characteristics as an important barrier for successful rehabilitation, which was not the case for ISH workers. These results may reflect a more protective and restraining attitude in RCFs than in ISH in line with current policies and guidelines, which state a higher potential of ISH interventions in the promotion of service users’ social inclusion and participation in the community (Gühne et al., 2019; United Nations, 2006; World Health Organization WHO, 2021).

Work-related satisfaction and health did not differ between the two settings. In ISH, MHCPs reported higher job demands, more opportunities for development, and greater influence at work than professionals in RCFs. Having influence and opportunities for development can contribute to employees’ satisfaction and health (Nübling et al., 2006). ISH therefore appears to be associated with more opportunities for independent, preference-based decision-making than RCFs, not only for service users (World Health Organization WHO, 2021) but also for MHCPs. In addition, MHCPs in both settings most frequently agreed with the statement that individuals with SMI best acquire housing skills when living independently with outreach support. Noteworthy, the majority of MHCPs working in RCFs agreed with this statement. This indicates that the traditional continuum approach is being increasingly evaluated critically not only in the literature (Fakhoury & Priebe, 2007; Priebe et al., 2005; Richter et al., 2016) but also during the practical everyday lives of housing rehabilitation professionals.

In both housing rehabilitation settings (ISH and RCFs), MHCPs showed remarkably high recovery orientation and open-mindedness toward individuals with SMI. This is of high relevance because the attitudes of MHCPs are crucial to the success of rehabilitation interventions (Richter et al., 2016). The importance of a strong recovery orientation of the supporting professionals was also emphasised by them in the qualitatively evaluated open questions. This, together with the availability of low-threshold support services, was highlighted by MHCPs in both settings as central factor in enabling persons with SMI to live in a stable and equitable manner.

Regarding obstacles to successful housing rehabilitation, however, MHCPs were faced with different problems between the settings. In RCFs, characteristics of service users (e.g. limited personal resources), of the support process (e.g. MHCPs’ lack of resources and flexibility regarding time and content), and contextual circumstances (especially the lack of affordable accommodations) were stated as obstructive factors with equal frequency. In ISH, on the other hand, characteristics of the service users do not seem to be an obstacle in the rehabilitation process. However, based on the available data, one cannot determine to what extent service users of the two settings differed in terms of their symptoms and functioning, despite the comparable indications of the services regarding service users’ level of impairment or functioning. Other research shows differences in diagnosis and gender of service users between different housing rehabilitation settings (de Heer-Wunderink et al., 2012; Dehn et al., 2022; Martinelli et al., 2019; Nordentoft et al., 2012). However, there is some evidence that questions a clear association between disease severity and the degree of institutionalisation (de Heer-Wunderink et al., 2012; Valdes-Stauber & Kilian, 2015). Thus, it remains a question of further research if service users in RCFs have actually more severe impairments, or if the view of service users’ non-readiness for independent living reflects the traditional continuum approach, which aims to first train and prepare service users before considering them competent to live independently. According to the surveyed MHCPs, outreach housing rehabilitation is mainly obstructed by social and environmental conditions. In particular, a lack in affordable accommodation, financial and regulatory hurdles and the social stigmatisation of individuals with SMI impede outreach housing rehabilitation and the social inclusion of service users.

The generalisation of the results might be limited. Data was collected only in one country and in a limited number of sites. In two of the three sites, the rehabilitation providing institutions each provide both settings and no fidelity assessment of the participating services was conducted. This could pose the risk that the institutions follow specific policies and do not explicitly follow normative guidelines of good services. Because the study was conducted as an online survey, generalisability may further be limited by participants’ self-selection. Despite the considerable response rate of 33%, participants may differ from non-participants, for example in their interest in the issue. In addition, response patterns might be biased toward social desirability.

The results of this study indicate that MHCPs’ preferences, attitudes or working conditions seem not to substantially contribute to the lagging implementation of the strongly preferred and widely recommended ISH interventions. MHCPs of both housing rehabilitation settings (ISH and RCFs) gave stronger endorsement of the ISH approach. However, the findings of this study show that societal and political conditions constitute major barriers. Therefore, successful housing rehabilitation in terms of an independent, self-determined life as a participating member of the community would be almost impossible for people with SMI without the support of housing rehabilitation professionals.

## Data Availability

The data that support the findings of this study are available from the corresponding author upon reasonable request.
